# Critical Quality Attribute Investigation and Analytical Method Establishment for Glyco-Site-Specific Anti-HER2 Bispecific ADC

**DOI:** 10.3390/ph19091495

**Published:** 2026-09-20

**Authors:** Xiaojuan Yu, Lele Wang, Weirong Cao, Yao Tang, Tao Tang, Yangwei Fu, Yalan Yang, Chuanfei Yu, Yan Li, Lan Wang

**Affiliations:** 1 National Institutes for Drug Control, State Key Laboratory of Drug Regulatory Science, NMPA Key Laboratory for Quality Research and Evaluation of Biological Products, NHC Key Laboratory of Research on Quality and Standardization of Biotech Products, Beijing 102629, China; yuxiaojuan@nifdc.org.cn (X.Y.); yangyalan@nifdc.org.cn (Y.Y.); yucf@nifdc.org.cn (C.Y.); 2 Jiangsu Alphamab Biopharmaceuticals Co., Ltd., Suzhou 215125, China; lelewang@alphamabonc.com (L.W.); yangweifu@alphamabonc.com (Y.F.); 3 CSPC Megalith Biopharmaceutical Co., Ltd., Shijiazhuang 050035, China; caoweirong@cspc.cn; 4 NMPA Center for Innovation and Research in Regulatory Science, Sichuan Institute for Drug Control, SCMPA Key Laboratory for Quality Monitoring and Risk Assessment of Biological Products, Chengdu 611731, China; tangyao_scidc@163.com (Y.T.); tangtao_scu@163.com (T.T.)

**Keywords:** bispecific antibody–drug conjugates, critical quality attributes, dual HER2 epitopes, quality control

## Abstract

**Background/Objectives**: Bispecific antibody–drug conjugates (bispecific ADCs) are among the most rapidly advancing antibody therapeutic modalities, with only one product (BL-B01D1) currently approved for marketing worldwide to date. In this study, we investigated several critical quality attributes (CQAs) of a glyco-site-specific antibody–drug conjugate (ADC) targeting two distinct epitopes of HER2. **Methods**: We established quality control methods for the assessment of mispaired species, conjugation site, drug-to-antibody ratios (DARs), charge variants, residual free drugs, residual β1,4-galactosyltransferase I (GalT) enzyme, relative binding activity, and cell proliferation inhibition activity. **Conclusions**: An analytical method suite was developed for the bispecific ADC to comprehensively characterize and appropriately control its critical quality attributes, thereby guiding the research, development, and manufacturing process optimization of the ADC product and ensuring its safety, efficacy, and quality consistency.

## 1. Introduction

Antibody–drug conjugates (ADCs) can precisely deliver toxic drugs to tumors. This strategy causes fewer side effects than chemotherapy [1]. However, their clinical application and efficacy improvements are still limited by three key bottlenecks: first, tumor cells have insufficient internalization capabilities for ADCs, and this limitation leads to the drugs being unable to effectively enter target cells and exert their effects [2]; second, there are nonspecific targeting effects that cause off-target toxicity to normal tissues and increase the risk of treatment-related adverse reactions [3]; third, tumor cells are prone to develop acquired resistance during treatment, and this leads to a decline in the drug efficacy as the treatment cycle extends [4]. Bispecific antibody–drug conjugates (BsADCs) combine ADCs with “dual-target experts” called bispecific antibodies (BsAbs) to address these challenges and achieve an anti-cancer effect of “1 + 1 > 2”. Compared to traditional monoclonal antibody–drug conjugates (mAb-ADCs), BsADCs have potential advantages that include the following: 1. a faster endocytosis rate than monoclonal antibody–drug conjugates; 2. enhanced binding to tumor cells that allow for the delivery of more payload, increasing the tumor-killing capability; and 3. a lower dependence on antigen expression levels, making the drug effective for patients with low antigen expressions [5].

The BsAb technology pathway has gained considerable clarity through recent research [6], and pharmaceutical companies around the world are further accelerating their research and development efforts. According to the Insight database, there are currently 19 bispecific ADC (BsADC) drugs that have entered clinical trials globally. Among them, only one—BL-B01D1, developed by Chinese company Baili-pharm—has been approved for marketing by the NMPA. Two other candidates are currently in Phase III clinical trials: JSKN003 [7], co-developed by CSPC Pharmaceutical Group and Alphamab, and TQB2102, developed by ZhenDa TianQing.

**Figure 1:**
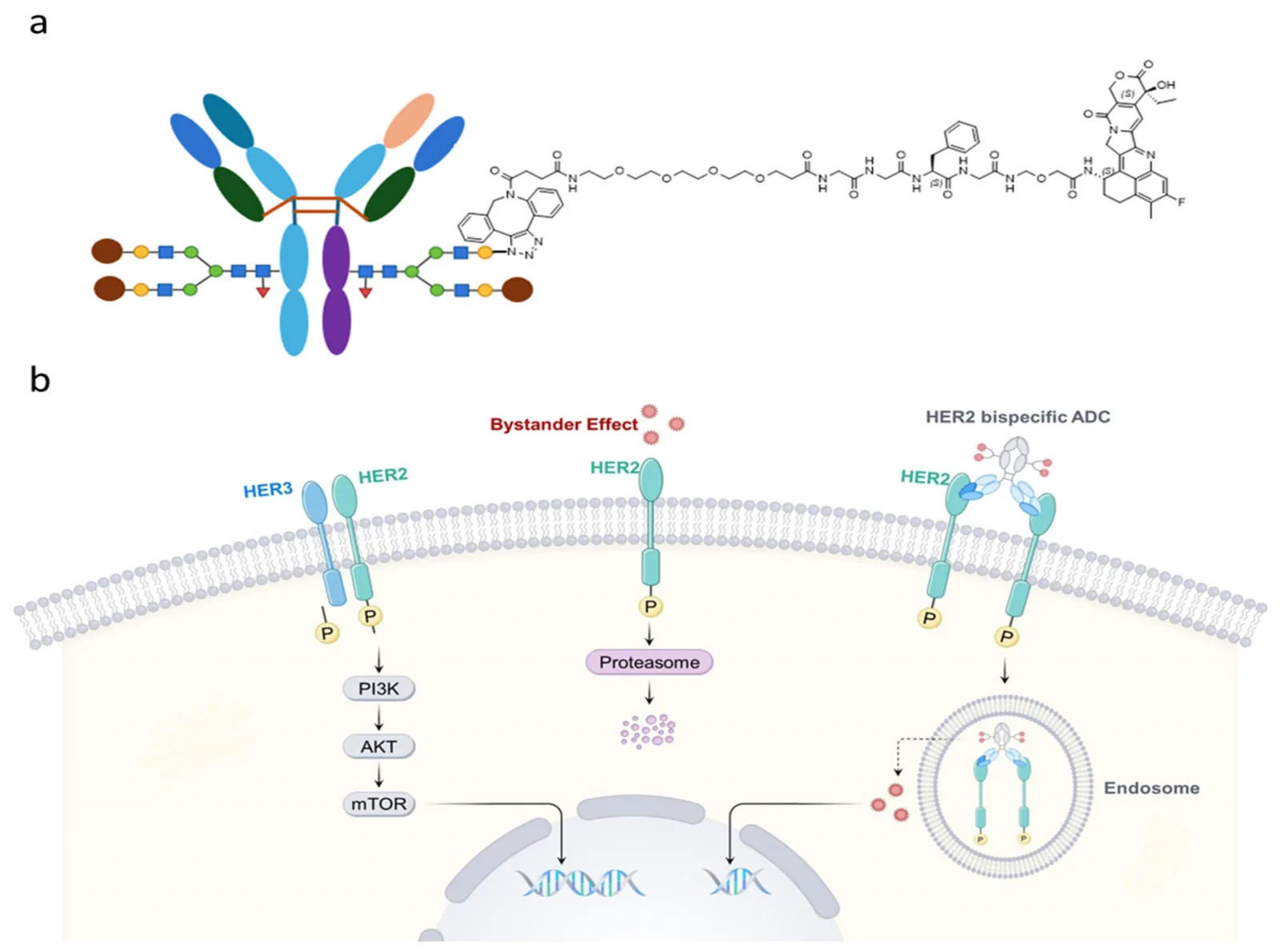
Schematic diagram of the molecular structure (**a**) and mechanism of action (**b**).

This study focuses on a HER2 biparatopic antibody–drug conjugate (ADC). The parental antibody is an IgG-type antibody targeting two distinct epitopes on HER2—domain II and domain IV of the extracellular region. The antibody moiety consists of a heterodimeric molecule comprising two different IgG1 heavy chains (Heavy Chain I and Heavy Chain II) and two identical kappa light chains, forming a common light-chain configuration. The payload is a topoisomerase I inhibitor, and the linker moiety comprises hydrophilic polyethylene glycol (PEG) units and a GGFG cleavage site. The glycans on the heavy chain were modified via a variant of the β-1,4-galactosyltransferase (GalT) variant [8], followed by click chemistry to yield the antibody–topoisomerase I inhibitor conjugate with a drug-to-antibody ratio (DAR) of approximately 4. A schematic representation of the molecular structure is provided in Figure 1a.

After binding to different epitopes of HER2 on tumor cell membranes, this HER2 bispecific ADC drug enters lysosomes via receptor-mediated endocytosis. In the lysosomes, the cleavable linker peptide of the ADC, GGFG, is hydrolyzed by proteolytic enzymes such as Cathepsin B, releasing the cytotoxic agent that causes DNA damage and induces tumor cell apoptosis. In addition, the cytotoxic agent can penetrate the cell membrane and kill tumor cells near the target cells through the bystander effect, further inhibiting tumor growth (See Figure 1b) [9,10].

Previous studies have demonstrated that the parental bispecific antibody exerts a “1 + 1 > 2” synergistic functional property in tumor cell models with varying HER2 expression levels [11]. Furthermore, this molecule, a glycan-specific conjugated ADC based on click chemistry, exhibits enhanced serum stability compared to conjugates based on maleimide–Michael reactions (e.g., Enhertu) [12]. The bispecific HER2 targeting provides the drug with stronger binding and endocytosis activity, leading to a stronger bystander killing effect [12]. Therefore, this bispecific ADC drug may have a wider therapeutic dose window in HER2-expressing solid tumors.

Currently, the published literature predominantly comprises preclinical studies; more systematic data on critical quality attributes (CQAs), control strategies, and analytical method validation for bispecific ADCs in the clinical stage remain to be reported in future studies. This study focuses on the aforementioned HER2 biparatopic ADC as the target product, aiming to investigate its critical quality attributes. Advanced analytical techniques including LC-MS, RP-HPLC, CEX-HPLC, ELISA, and cell proliferation inhibition assays were employed to establish key quality control methods for this class of ADC products. These methods encompass the detection of bispecific ADC-specific attributes such as mispaired species, conjugation sites, DAR, charge variants, residual free drugs, residual GalT enzyme, relative binding activity, and cell-proliferation-inhibitory activity. This work is intended to serve as a reference for quality research and batch release control of this category of ADC [13].

## 2. Results

### 2.1. Mispairing

The unconjugated antibody of this product is a heterodimeric antibody molecule composed of two different IgG1 subtype heavy chains: Heavy Chain I, Heavy Chain II, and two identical kappa subtype light chains (common light chain). To improve the resolution of the reverse-phase method, the sample was digested using the Ides enzyme. The unconjugated antibody was divided into F(ab′)2 and Fc/2 parts, and the half-antibody and mispaired homodimer were divided into corresponding F(ab′) and F(ab′)2 parts; then, in the sample, the mispaired F(ab′)2 and half-antibody F(ab′) were effectively separated using RP-HPLC. The percentage content of the mispaired components in the sample was calculated using the area normalization method.

**Figure 2:**
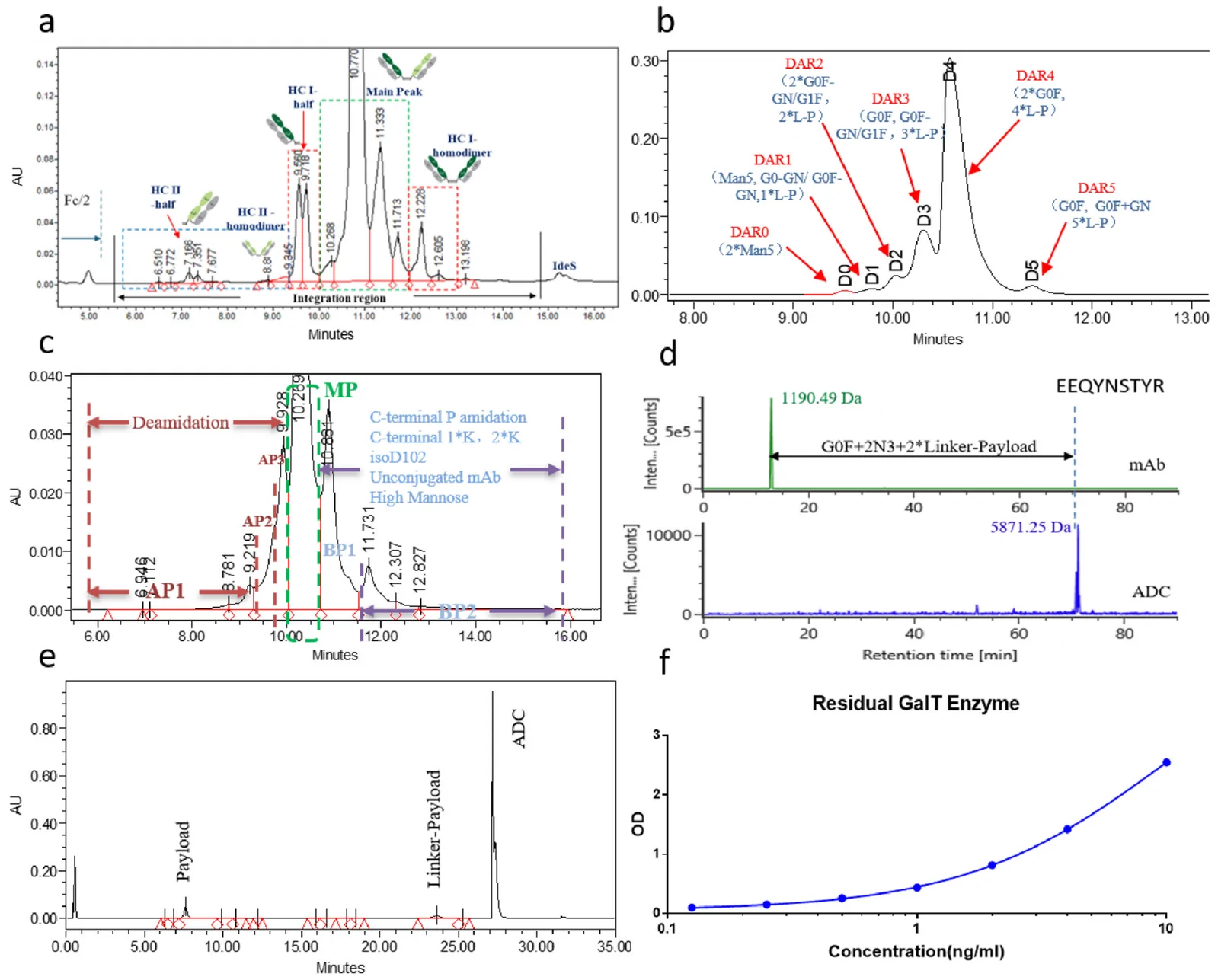
Typical spectra of the method. (**a**) Mispairing method spectrum and peak identification results of the unconjugated antibody (Fc/2 represents the Fc segment of the unconjugated antibody sample after IdeS digestion, HCII−half represents the F(ab′) portion of Heavy Chain II after IdeS digestion, HCII−homodimer represents the F(ab′)2 portion of the mispaired Heavy Chain II after IdeS digestion, HCI−half represents the F(ab′) portion of Heavy Chain I after IdeS digestion, and the HCI−homodimer represents the F(ab′)2 portion of the mispaired Heavy Chain I after IdeS digestion). The green box indicates the correctly paired main component, the red box indicates the peak positions of the HCI−half and HCI−homodimer, and the blue box indicates the peak positions of the HCII−half and HCII−homodimer. (**b**) DAR spectrum and peak identification results. (**c**) Charge variants chromatogram, component collection areas, and identification results. (**d**) Mass spectra of glycopeptides before and after deglycosylation. (**e**) Chromatogram and identification results of the free drugs. (**f**) Four-parameter fitting standard curve of the GalT enzyme.

The mass spectrometric analysis results of this method showed that the set of peaks with retention times of 6–8 min were the half-antibody of Heavy Chain II, with a molecular weight of 48,816 Da. The peak with a retention time of 9.3 min was the homodimer of Heavy Chain II, with a molecular weight of 97,624 Da. The two peaks with retention times of 9.4–10 min were the half-antibody of Heavy Chain I, with a molecular weight of 48,747 Da. The set of peaks with retention times 10–12 min was the primary component of the correctly paired antibody (Figure 2a, green box), with a molecular weight of 97,562 Da. The set of peaks with retention times of 12–13 min was the homodimer of Heavy Chain I, with a molecular weight of 97,494 Da. Therefore, in addition to the homodimeric F(ab′)2 form, the homodimeric F(ab′) form also existed in the spectrum. The impurity of Heavy Chain I was reported as the sum of the peak area percentages of the half-antibody of Heavy Chain I and the homodimer of Heavy Chain I (Figure 2a, red box). The impurity of Heavy Chain II was reported as the sum of the peak area percentages of the half-antibody of Heavy Chain II and the homodimer of Heavy Chain II (Figure 2a, blue box). The impurity of Heavy Chain I in this product was (0.8 ± 0.09)%, and the impurity of Heavy Chain II was <0.5%.

The method was validated in accordance with the ICH Q2 guidelines. The validation results, as summarized in Table S1, demonstrated satisfactory specificity, robustness, linearity, accuracy, precision, and LOQ, confirming that the method is fit for its intended purpose.

### 2.2. Average DAR, Unconjugated Antibody, and Drug Conjugation Distribution

This molecule is an N-glycosylation site-specific conjugated ADC on the Fc region, with intact inter-chain disulfide bonds. Reverse-phase chromatography with a polymer resin showed six peaks from DAR0 to DAR5 that can be sequentially separated within an appropriate elution gradient (Figure 2b). The average DAR, unconjugated antibody content (DAR0), and drug distribution can be simultaneously calculated. The equation for calculating the average DAR is

$$\frac{A_{D0}\times 0+A_{D1}\times 1+A_{D2}\times 2+A_{D3}\times 3+A_{D4}\times 4+A_{D5}\times 5}{A_{D0}+A_{D1}+A_{D2}+A_{D3}+A_{D4}+A_{D5}}.$$

The DAR-method chromatography column was split and connected to the mass spectrometer. The peak in the DAR spectrum was identified by the intact mass spectrum. DAR0 represents the unconjugated antibody content, with a primary-component molecular weight of 147,355 Da. DAR1–DAR5 represent the ADC components conjugated with 1–5 linker–payloads, respectively.

Using this method, the unconjugated antibody content of this product was (0.8 ± 0.04)%, the primary component DAR4 content was (77.3 ± 1.0)%, and the average DAR was 3.7 ± 0.03. The validation results, as summarized in Table S2, demonstrated satisfactory specificity, robustness, linearity, accuracy, precision, and LOQ.

### 2.3. Charge Variants

Based on the hydrophilicity of the topoisomerase I inhibitor and the PEG4 linker, the weak cation exchange column from Thermo Fisher Scientific was selected. The results showed minimal nonspecific secondary interactions with this ADC molecule that can be used for a charge-variant study. The spectrum of the ADC charge-variant method is shown in Figure 2c. According to the cation exchange chromatography separation mechanism, the acidic species, primary peak, and basic species were eluted sequentially between 7 and 16 min. The resolution between the acidic species, primary peak, and basic species was good. The use of this method showed that the acidic species content of this product was (14.9 ± 0.7)%, the primary peak content was (65.0 ± 0.7)%, and the basic species content was (20.1 ± 1.0)%. The validation results, as summarized in Table S3, demonstrate satisfactory specificity, robustness, linearity, accuracy, precision, and LOQ, confirming that the method is fit for its intended purpose.

Peak collection identification: the acidic species were divided into three peak collection regions: the main peak and the basic species were divided into two peak collection regions for peak collection (see Figure 2c for the purity of each collected fraction; the reinjection chromatograms are shown in Table S4 and Figure S1). The six collected fractions were analyzed for the following physicochemical properties: the DAR, molecular weight, post-translational modifications, thermal stability (DSF), biological functions, relative binding activity, cell proliferation inhibition activity, and Fc receptor affinity (FcRn, FcγRIIIa (V176 and F176), and C1q). The peak collection identification results are shown in Table 1. These results show the following: ① The acidic species primarily had a higher proportion of deamidation modifications (light chain N30 and heavy chain N55). The basic species primarily had a higher proportion of C-terminal P amidation, a higher proportion of C-terminal K variants, and a higher proportion of heavy-chain D102 isomerization. In addition, the basic region exhibited slightly higher levels of Man5 glycoform and unconjugated antibody, as determined by molecular weight analysis, along with a lower DAR (3.5) relative to the main peak (3.8). ② The relative binding activity, proliferation inhibition activity, and Fc receptor affinity of the primary peak, acidic species, and basic species showed no significant differences compared to the sample before collection.

### 2.4. Conjugation Site

This product utilizes site-specific glycosylation technology. The coupling site for the linker–payload is the N-glycosylation site in the Fc region of this product—specifically, heavy chain I N299/heavy chain II N300. The theoretical N-glycosylation site (coupling site) is located in the EEQYNSTYR peptide. The mass spectra of the peptide before and after deglycosylation are shown in Figure S2. In the deglycosylated sample, this peptide showed deamidation modification, with a measured molecular weight of 1190.49 Da and an observed *m*/*z* of 595.7479 (z = 2+). In the untreated sample, the peptides that contained the N-glycosylation site all showed glycosylation and conjugated linker-payload. The measured molecular weight of this coupled peptide was 5871.25 Da, and the observed *m*/*z* was 1175.0515 (z = 5+). The deviation between the measured molecular weight and the theoretical molecular weight of each peptide was less than 20 ppm (See Table S5); the mass spectra of glycopeptides before and after deglycosylation are shown in Figure 2d. These results demonstrate that the conjugation sites were consistent with the theoretical predictions. A high conjugation occupancy of 99.1% was achieved at the site-specific glycan conjugation sites, demonstrating the exceptional efficiency of the conjugation strategy (See Table S6).

### 2.5. Free Drugs

A reversed-phase chromatography method employing an Agilent PLRP polymer-based column (1000 Å pore size, phenyl stationary phase) was utilized, taking advantage of its suitability for macromolecule separation to simultaneously resolve Payload, Linker-Payload, and ADC molecules. The residual free payload was quantified by external standard calibration using Payload and Linker-Payload standards. A representative chromatogram obtained by this method is shown in Figure 2e. The ADC main component eluted at a retention time of 27–28 min, with the Payload peak appearing at a relative retention time (RRT) of 0.3 and the Linker-Payload peak at an RRT of 0.9 relative to the ADC main peak. Baseline separation was achieved between Payload and Linker-Payload. The method was applied to the analysis of multiple clinical batch samples, and the residual Payload levels were found to be below the LOQ (0.01%) in all batches, while the Linker-Payload residual levels were determined to be (0.04 ± 0.02)%. The validation results, as summarized in Table S7, demonstrated satisfactory specificity, robustness, linearity, accuracy, precision, and LOQ.

**Figure 3:**
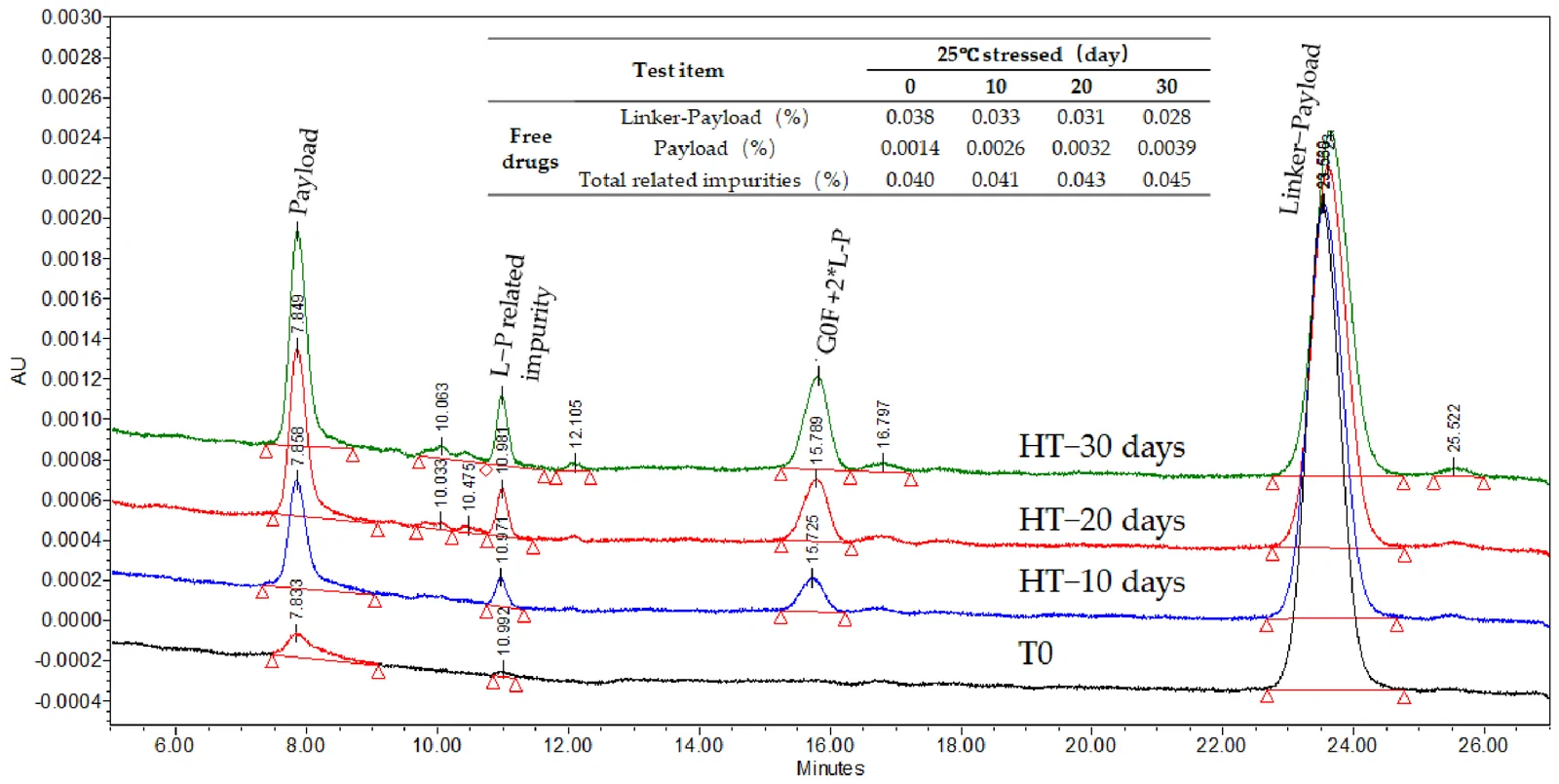
Chromatographic profiles and partial characterization of the free drugs under high-temperature stress conditions (“L-P” in the figure stands for Linker-Payload; to illustrate the variation trends of Payload and Linker-Payload residues under stress conditions, the data are presented with two significant figures). T0, HT-10 days, HT-20 days and HT-30 days, correspond to T0, 25 °C high-temperature stress for 10 days, 20 days, and 30 days, respectively.

Under stressed conditions at 25 °C for 30 days, new drug-related impurity peaks were observed (Figure 3). Based on LC–MS/MS identification, the newly appearing peak at a retention time of 15.8 min was assigned as G0F + 2 (Linker + Payload), corresponding to two Linker + Payload moieties conjugated to the terminal N-glycans at heavy chain I N299 and heavy chain II N300. These N-glycan-linked conjugates were found to be labile under elevated temperature, resulting in the release of the Linker + Payload adducts. In addition, a major impurity peak at 11.0 min was characterized by MS and MS/MS analysis, with a measured molecular ion [M + H]^+^ of *m*/*z* 1188.4951. This species shared several fragment ions with the Linker + Payload standard, suggesting that it is a related degradation product of the Linker + Payload. Compared with the T0 time point, the payload level exhibited an upward trend after 30 days at 25 °C, whereas the Linker + Payload level showed a declining trend. The total level of related impurities remained essentially unchanged, and the recovery was 112.5%.

### 2.6. Residual GalT Enzyme

A double-antibody sandwich enzyme-linked immunosorbent assay (ELISA) was employed for the sensitive determination of GalT enzyme residues in ADC samples. The limit of quantification (LOQ) of the method was established at 0.05 ppm. Calibration was performed using a four-parameter logistic (4-PL) curve fit relating GalT enzyme content to absorbance values, with the standard curve (ranging from 0.25 to 5.0 ng/mL) presented in Figure 2f. The method exhibited good performance in terms of accuracy, linearity, precision and LOQ (see Table S8).

### 2.7. Relative Binding Activity

**Figure 4:**
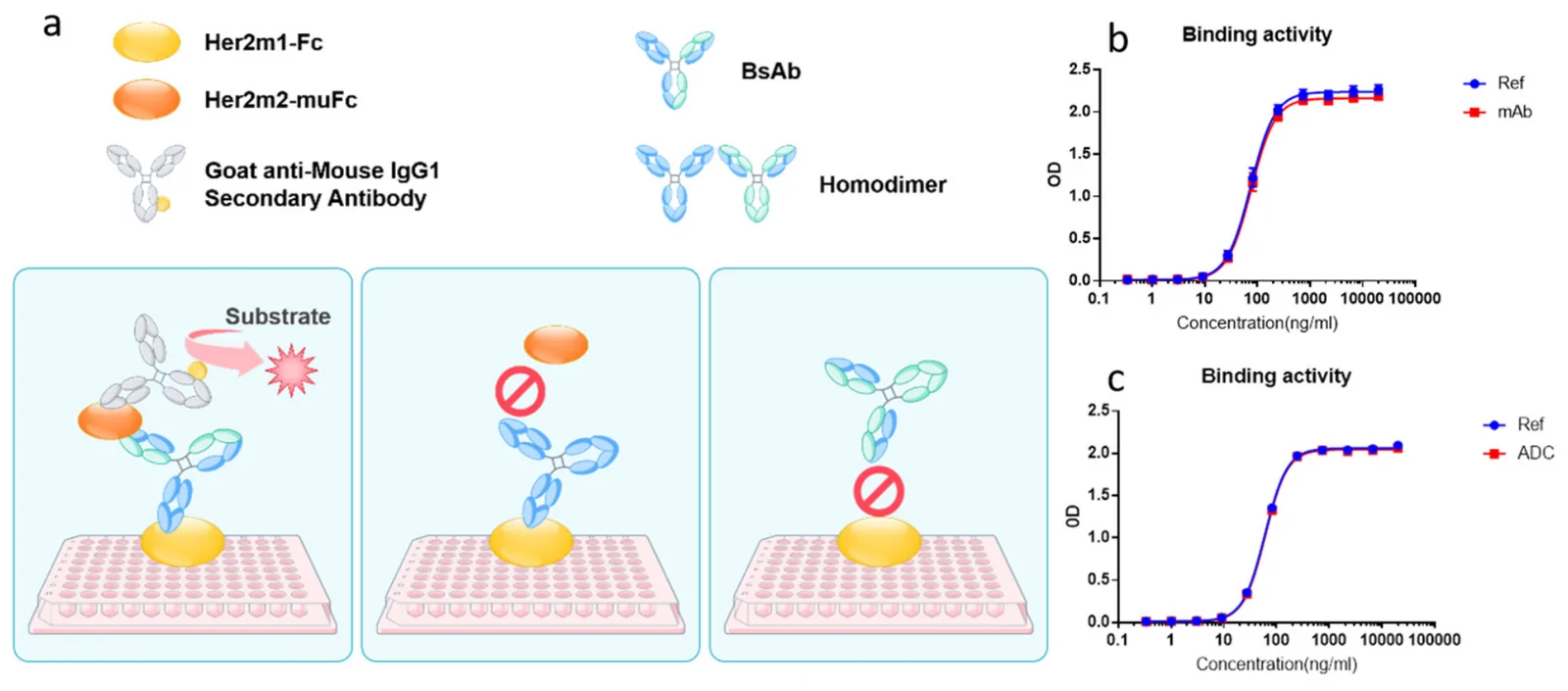
Relative binding activity. (**a**) Schematic diagram; (**b**) four-parameter fitting curve results for unconjugated antibody, *n* = 6, $\bar{X}$ ± s; (**c**). four-parameter fitting curve results for ADC, *n* = 6, $\bar{X}$ ± s.

The binding activities of the unconjugated antibody/ADC to distinct epitopes of the HER2 antigen were determined using a bridging ELISA (see Figure 4a). In all six replicate assays, a clear dose–response relationship was observed for both the unconjugated antibody (Figure 4b) and the ADC (Figure 4c), exhibiting typical sigmoidal curves, with correlation coefficients exceeding 0.99 in all cases. The mean relative binding activity of the unconjugated antibody was (93 ± 0.9)%, with an RSD of less than 1%. The mean EC_50_ values of the unconjugated antibody were (76.46 ± 0.860) ng/mL. For the ADC, the mean relative binding activity was (97 ± 2.3)%, with an RSD of less than 3%. The mean EC_50_ values of the ADC were (61.28 ± 0.473) ng/mL. The method exhibited good performance in terms of accuracy, linearity, and precision within the tested range of 50–201% (see Table S9).

### 2.8. Biological Activity

**Figure 5:**
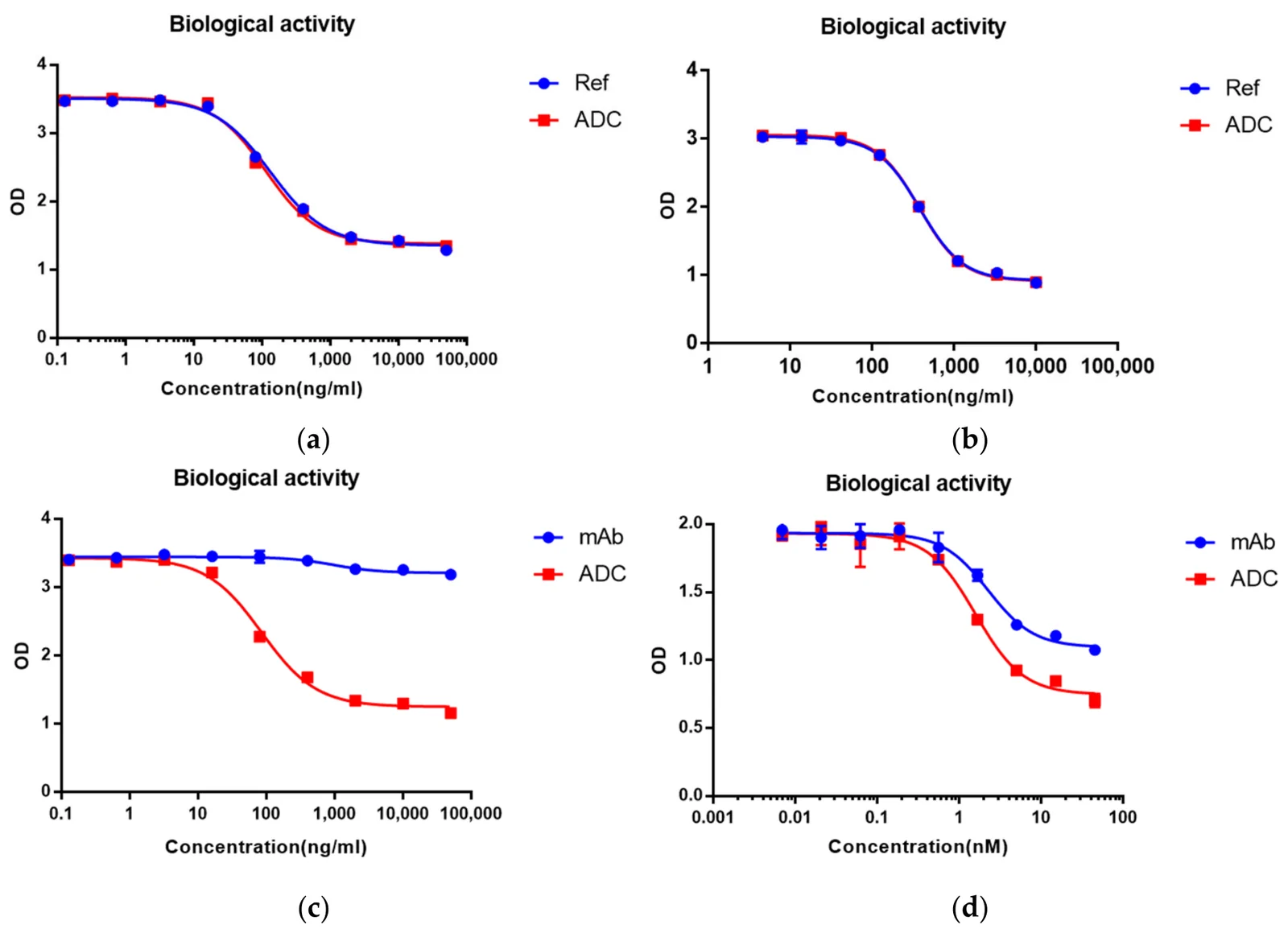
Cell proliferation inhibition activity. (**a**) Four-parameter logistic (4PL) fit curves for HCC1569 target cells (*n* = 3); (**b**) four-parameter logistic (4PL) fit curves for NCI-N87 target cells (*n* = 3); (**c**) four-parameter logistic (4PL) fit curves for cell proliferation inhibition by unconjugated antibody and ADC in HCC1569 target cells; (**d**) four-parameter logistic (4PL) fit curves for cell proliferation inhibition by unconjugated antibody and ADC in NCI-N87 target cells.

The cell proliferation inhibition assay using HCC1569 as target cell showed dose–response relationships in all three tests, presenting typical reverse S-shaped curves (Figure 5a), with correlation coefficients all greater than 0.99. The mean value of the relative activity of the sample relative to the reference substance was (109 ± 9.6)%, with an RSD of <9%. The mean IC_50_ value of the ADC was determined to be (112.2 ± 8.176) ng/mL. The method exhibited good performance in terms of accuracy, linearity, and precision within the tested range of 50–201% (see Table S10).

The cell proliferation inhibition assay using NCI-N87 as target cell showed dose–response relationships in all three tests, presenting typical reverse S-shaped curves (Figure 5b), with correlation coefficients all greater than 0.99. The mean value of the relative activity of the sample relative to the reference substance was (98 ± 2.0)%, with an RSD of <3%. The mean IC_50_ value of the ADC was calculated to be (377.2 ± 12.31) ng/mL.

Comparison of the proliferation inhibition activity between the unconjugated antibody and ADC sample is shown in Figure 5c,d. The figure shows that in the system with HCC1569 as target cell, the unconjugated antibody contributes little to the proliferation inhibition activity, while in the system with N87 as target cell, the unconjugated antibody contributed a considerable part of proliferation inhibition activity. In the NCI-N87 cell model, the ADC may retain the HER2 signal-blocking activity conferred by its parental antibody, which mediates the inhibition of proliferation in HER2-expressing tumor cells.

### 2.9. CQA Assessment

The assessment of product-critical quality attributes uses the risk priority number (RPN) method, which evaluates the “Severity” and “Likelihood” of impact for each attribute. The RPN formula is: Critical Attribute RPN = Severity × Likelihood [14].

The severity assessment considers both safety (e.g., toxicity and immunogenicity) and product efficacy (e.g., activity and pharmacokinetics/pharmacodynamics). It includes known and potential aspects that can be based on product-specific knowledge, platform knowledge, or prior knowledge. The likelihood is defined as the probability that an adverse event that affects safety and/or efficacy will occur due to quality attributes that exceed the range specified based on the current knowledge space. The knowledge space is established based on clinical and nonclinical studies of this molecule or similar molecules, as well as the relevant literature. When clinical data on the impact of a specific quality attribute on safety and/or efficacy are limited, a conservative score (≥5) should be assigned.

The criticality scoring of attributes may decrease as knowledge is gained during the product life cycle (typically reflected in reduced likelihood scores) [15].

**Figure 6:**
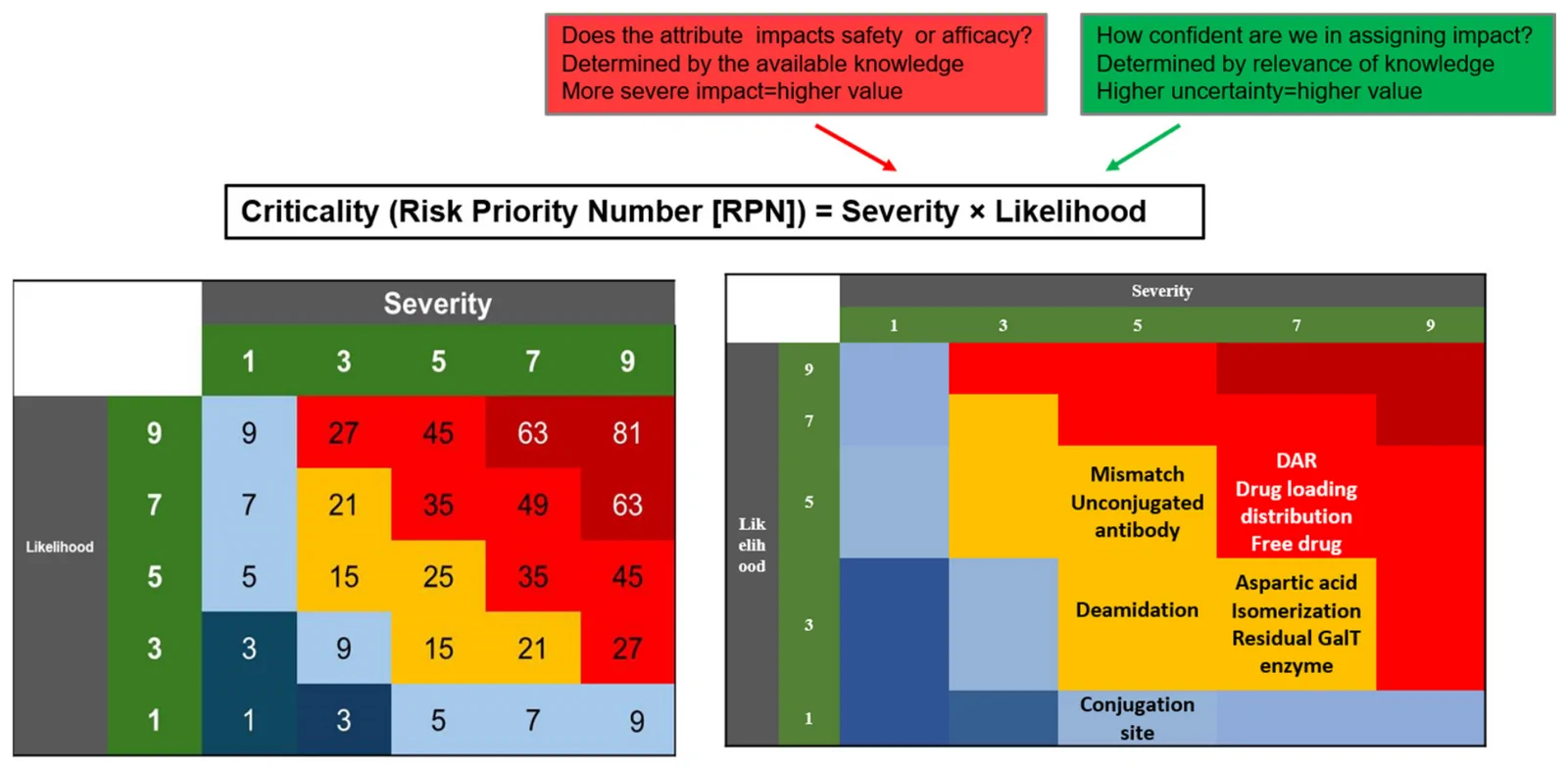
CQA assessment tool and risk score defined by severity and likelihood. Product quality attributes with risk scores of 1–9 were classified as low-risk quality attributes (blue boxes), those with scores of 15–25 as medium-risk quality attributes (yellow boxes), and those with scores of 27–81 as high-risk quality attributes, i.e., critical quality attributes of the product (red boxes).

The risk assessment process and scoring for the aforementioned approaches, based on Severity and Likelihood, are presented in Table 2. To facilitate the classification of risk levels for each quality attribute, the corresponding risk matrix is graphically illustrated in Figure 6.

## 3. Discussion

For the asymmetric bispecific ADC, mispairing is a critical quality attribute that needs to be controlled for the molecular design and manufacturing process. The mispairing control strategy for this molecule included the following: (1) Molecular design: an amino acid mutation was used to prevent large-scale mispairing [13]. (2) Manufacturing process: the upstream development of the cell culture process with a low mispaired content; the downstream hydrophobic interaction chromatography can effectively remove mispairings [24]; the multi-batch manufacturing process was stable, and the mispaired impurities were effectively controlled. (3) The antibody–drug conjugates (ADCs), with a drug-to-antibody ratio (DAR) of ~4, were prepared via enzymatic modification of the native heavy-chain glycans on the antibody using a β-1,4-galactosyltransferase (GalT) variant, followed by click conjugation. Since no disulfide bond reduction is involved in these steps, they do not introduce any additional impact on the mispairing species already present in the antibody. (4) Quality control: considering that the mispaired impurity purity is already monitored during unconjugated antibody release, the statistical results of the 24 batches of unconjugated antibody drug substance release during the clinical period showed that the mispaired impurity purity met the specification, and the stability testing results of the unconjugated antibody intermediate within the shelf life showed no significant change trend of the mispaired impurities. Therefore, mispairing is not required to be controlled in the ADC drug substance and drug product.

DAR is a critical quality attribute of ADCs. A lower ratio reduces the efficacy, while a higher ratio poses potential risks to safety and stability. Controlling the DAR ensures consistency in batch-to-batch production of antibody–drug conjugates, and its measurement can provide feedback for process control, which is crucial in optimizing the process parameters. The uniformity of DAR also indicates good controllability of the efficacy and safety [25]. This reverse-phase method can simultaneously determine the average DAR, the content of the unconjugated antibody (DAR0), and the drug conjugation distribution. It is of significant reference value for the analysis of DAR and the drug distribution in ADCs based on intact disulfide bonds after conjugation. Based on the N-glycan profile of the unconjugated antibody, glycans (FA2 + A2) are amenable to dual linker–payload conjugation via GalT1-mediated azide introduction followed by strain-promoted azide–alkyne cycloaddition (SPAAC), whereas glycans (A1 + FA1 + FA2G1) permit only single-site conjugation and glycans (A2G2 + FA2G2 + FA2G2S1 + FA2G2S2) are refractory to conjugation. Mass spectrometry (MS) analysis revealed that the predominant component of the DAR0 peak had a molecular mass of 147,355 Da, corresponding to the Man5/Man5 glycoform, which is incapable of linker–payload conjugation. The DAR1–DAR5 peaks represent ADC species conjugated with one to five linker–payload moieties, respectively. Specifically, the major component of the DAR1 peak was identified as the Man5/G0F-GN glycoform, with a molecular mass of 149,001 Da, capable of conjugating one linker–payload, and was therefore designated as DAR1. The major component of the DAR2 peak exhibited a molecular mass of 151,377 Da, corresponding to the 2×G1F glycoform, which supports conjugation of two linker–payload moieties, and was therefore designated as DAR2. The DAR3 peak showed a predominant component of 152,834 Da, assigned to the G0F/G1F glycoform, capable of conjugating three linker–payload entities, and was designated as DAR3. The DAR4 peak had a major component of 154,293 Da, corresponding to the 2×G0F glycoform, which enables conjugation of four linker–payload moieties, and was therefore designated as DAR4. The DAR5 peak displayed a predominant component of 156,113 Da, assigned to the G0F+GN/G1F glycoform, capable of conjugating five linker–payload moieties, and was designated as DAR5. Therefore, the DAR can be calculated using a weighted formula: (G0F-related glycoform proportion × 2 + G1F-related glycoform proportion × 1 + G2F-related glycoform proportion × 0) × 2 ÷ 100). The glycan proportions in the N-glycan profile of the unconjugated antibody directly affect the DAR, allowing the DAR after conjugation to be predicted during the cell culture stage.

Charge-variant analysis of therapeutic proteins is commonly performed using ion-exchange chromatography (IEX), capillary zone electrophoresis (CZE), or capillary isoelectric focusing (cIEF). However, the development of robust IEX or imaged capillary isoelectric focusing (iCIEF) methods for antibody–drug conjugates (ADCs) is particularly challenging [26], owing to the increased hydrophobicity imparted by the payload–linker, the inherent heterogeneity of the drug-to-antibody ratio (DAR), and the instability of the lactone ring structure within the payload. Compared with the iCIEF approach, the CEX-based release method offers a distinct practical advantage, as it facilitates more convenient fraction collection, peak identification, and subsequent functional characterization. In this study, peak fraction identification revealed that the acidic region was predominantly composed of highly deamidated species. In addition to the commonly observed high levels of C-terminal proline amidation and C-terminal lysine variants in the basic region, our results further demonstrated that the basic region contained a notable proportion of heavy-chain D102 isomerization, along with moderately elevated levels of the Man5 glycoform and unconjugated antibody. Mass spectrometric analysis of the DAR profile confirmed that the D0 peak corresponded to the Man5 glycoform attached to both heavy chains in combination with unconjugated antibody. Collectively, these findings indicate that the slightly lower DAR observed in the basic region (DAR = 3.5) relative to the main peak (DAR = 3.8) is attributable to the modestly increased proportions of the Man5 glycoform and unconjugated antibody. Importantly, no significant differences were observed among the acidic, main, and basic fractions with respect to relative binding activity, cell proliferation inhibition activity, or Fc receptor affinity, suggesting that the DAR difference between 3.5 and 3.8 does not translate into a measurable impact on in vitro bioactivity. As our understanding of this molecule has advanced, charge variants have ultimately been classified as a non-critical quality attribute. Nevertheless, given that the relative proportions of acidic, main, and basic peaks are influenced by post-translational modifications and DAR, this CEX method remains suitable as a release assay for the monitoring of process consistency and batch-to-batch stability.

For the glycan-site-specific conjugation technology platform, the conjugation sites are theoretically the sites of N-glycosylation. The peptide fragment that contained the N-glycosylation site (conjugation site) was EEQYNSTYR, and the theoretical molecular weight of the non-glycosylated form is 1189.51 Da. In results without the deglycosylation treatment, the peptide fragment that contained the N-glycosylation site was found to exhibit both glycosylation and conjugation of linker-payload. The modification was G0F + 2 GalNAz + 2 linker–payload. The molecular weight of the linker–payload residue is 1374.56 Da, and that of the GalNAz residue is 244.08 Da. The molecular weight of the G0F was 1444.53 Da. The theoretical molecular weight of the conjugated peptide was therefore calculated to be 5871.32 Da.

The purification or ultrafiltration/diafiltration step after the conjugation reaction is a critical step for removing the free payload. Therefore, during CMC process development and drug production, it is necessary to develop an appropriate method to accurately quantify the free drugs. Currently, a commonly used method involves precipitating proteins (including the payload conjugated on the ADC molecule), then detecting the residual free payload in the supernatant. However, the difficulty in developing this method lies in the fact that when organic solvents or high levels of salt are added to precipitate the proteins, the free payload, due to its inherent hydrophobicity, may interact with the ADC and co-precipitate, thereby reducing the method’s recovery rate [27]. The free payload detection method discussed in this article utilizes a phenyl stationary phase in a polymer matrix that is suitable for the detection of large molecules. This method can simultaneously separate the payload, linker–payload, and ADC, reducing sample pretreatment steps and avoiding the risk of low recovery of free drugs [28].

In this process, β-1,4-galactosyltransferase (GalT) can transfer GalNAz from the substrate, UDP-GalNAz, to the terminal, GlcNAc, of the N-glycan on the heavy chain of the unconjugated antibody. As a host cell protein in Chinese hamster ovary (CHO) cells [29], the analytical method for residual GalT quantification was designed based on the same principle as the HCP detection assay platform. A sandwich ELISA can sensitively detect the residual GalT enzyme levels in ADC samples. Using this method, the measured residual GalT enzyme levels in multiple clinical batches were all below 2 ppm, which is significantly lower than the ADC drug substance specification limit of 20 ppm.

The parental antibody is a HER2 bispecific antibody capable of simultaneously binding to two non-overlapping epitopes of HER2, thereby blocking downstream HER2 signaling. For the relative binding activity assessment, a bridging ELISA was designed, in which the unconjugated antibody and its ADC simultaneously bind to both HER2m1 and HER2m2 antigens, forming an antigen–antibody–antigen bridging complex. As evidenced by the binding curves and EC_50_ values, the ADC exhibited binding affinity comparable to that of its unconjugated antibody counterpart, indicating that the chemical conjugation process did not impair the antibody’s ability to recognize and bind HER2. This method is employed as a release assay for confirming the binding activity of the bispecific antibody and the corresponding ADC to distinct HER2 epitopes and is therefore considered a critical quality attribute (CQA) of the product.

For cell proliferation inhibition assays, HCC1569 and NCI-N87 cells were employed as target cells, both of which naturally overexpress HER2 and are derived from human breast and gastric carcinomas, respectively. In this assay, the ADC binds to distinct domains of the HER2 antigen on the cell surface, mediating internalization and subsequent intracellular release of the small-molecule payload, leading to tumor cell killing. This method directly reflects the primary mechanism of action (MoA) of the ADC molecule and was therefore designated as a critical quality attribute (CQA). The cytotoxicity curves revealed differential sensitivity of the two tumor cell lines to both the unconjugated antibody and the ADC. In the HCC1569 target cell system, the cytotoxic activity is predominantly attributable to the toxin payload, with the HER2 signaling-pathway inhibition mediated by the antibody Fab region contributing minimally to the overall activity. In contrast, in the NCI-N87 target cell system, the HER2 signaling blockade by the Fab region contributes substantially to the observed activity. Notably, in addition to direct cytotoxicity, the ADC appears to retain the HER2 signaling-inhibitory function inherited from its parental antibody, thereby suppressing the proliferation of HER2-expressing tumor cells.

Our previous studies have demonstrated that, in the NCI-N87 CDX model, the ADC exhibits significantly greater tumor growth inhibition compared with the parental antibody [12], with a more pronounced difference in antitumor efficacy between the two treatments. This disparity may be attributed to the differential drug accessibility within the tumor microenvironment. In vitro cytotoxicity assays are performed on monolayer cell cultures where drugs can fully contact target cells, whereas in CDX models, tumors grow as three-dimensional solid masses, limiting antibody penetration into the tumor interior. The ADC, however, can exert a bystander effect through the release of its membrane-permeable payload, thereby achieving superior tumor growth suppression. Indeed, the spatial heterogeneity within solid tumors profoundly influences the therapeutic efficacy of ADC drugs. Nevertheless, in vitro cytotoxicity assays remain a robust and reliable method for routine potency release testing of ADC products. Between the two cell lines, we selected the HCC1569-based assay system as the release method for our ADC product, as it provides better discrimination of payload-mediated cytotoxic activity and more effectively reflects the primary MoA of the ADC.

Bispecific antibody–drug conjugates (bispecific ADCs), leveraging their unique mechanisms of dual-epitope cooperative recognition and precise delivery of highly cytotoxic payloads, represent a pivotal direction for the next generation of ADC therapeutics. However, the inherent structural complexity of bispecific ADCs far exceeds that of conventional monospecific ADCs, posing unprecedented challenges for quality attribute studies. In addition to the aforementioned quality attributes, the quality control of bispecific ADCs should also comprehensively consider size heterogeneity, process-related impurities (e.g., residual organic solvents and reaction by-products), Fc receptor-mediated functions (such as ADCC and CDC), and higher-order structure, all of which are relevant to product safety and efficacy. These factors collectively impose significant burdens on the development of analytical methods and the formulation of quality control strategies.

In this context, quality attribute studies permeate the entire lifecycle of bispecific ADC development, serving as the “eyes of process development”—sensitively illuminating the impact of process variations on product structure, modification, and activity, thereby facilitating accelerated development timelines and promoting clinical translation. Concurrently, quality attribute studies constitute an “essential cornerstone of release quality control”—by establishing scientifically rigorous analytical methods and appropriate acceptance criteria, they ensure batch-to-batch consistency and stability, thereby erecting a robust barrier for product safety, efficacy, and quality controllability. In this study, we have preliminarily established an analytical methodological framework for the assessment of critical quality attributes (CQAs) of a glyco-site-specific bispecific ADC targeting dual HER2 epitopes, encompassing multiple dimensions including mispaired species, conjugation sites, the drug-to-antibody ratio (DAR), charge variants, residual impurities, and biological activities. This systematic approach provides a reference for the quality research and process optimization of this class of products.

## 4. Materials and Methods

### 4.1. Sample

The unconjugated antibody is an anti-HER2 dual-epitope antibody expressed by Chinese hamster ovary (CHO) cells and processed into an intermediate through chromatographic purification and viral clearance. Subsequently, an ADC with a DAR of approximately 4 was generated by introducing azide groups onto the antibody N-glycans and conjugating with a dibenzocyclo-octyne-tetrapeptide linked to a topoisomerase I inhibitor. Both the ADC and the reference standard were prepared in-house, with retained samples kept at the National Institutes for Food and Drug Control (NIFDC), Beijing, China.

### 4.2. Primary Instruments and Reagents

Primary instruments included a UPLC Acuity Premier (Waters Corporation, Milford, MA, USA), an HPLC Arc Premier (Waters), an Waters I-Class UPLC system coupled with a Waters Xevo G2-XS QTof high-resolution mass spectrometer, and a SpectraMax M5e microplate reader (Molecular Devices, LLC, San Jose, CA, USA).

A reversed-phase MabPac RP 4 μm chromatographic column (2.1 × 150 mm, Thermo Fisher Scientific, Waltham, MA, USA), reversed-phase BioCore RP-1000 chromatographic column (5 μm, 3.0 × 150 mm, NanoChrom Technologies, Suzhou, China), a ProPac^TM^ Elite WCX column (5 μm, 4 × 150 mm, Thermo Fisher), a reversed-phase PLRP-S chromatographic column (2.1 × 50 mm, 5 um, Agilent Technologies, Santa Clara, CA, USA), a reversed-phase ACQUITY PREMIER Peptide CSH C18 chromatographic column (130 Å, 1.7 um, 2.1 × 150 mm, Waters), the IdeS enzyme (RhinoBio, Suzhou, China), chromatographic-grade acetonitrile (Fisher Scientific, Pittsburgh, PA, USA), mass spectrometry-grade acetonitrile (Merck KGaA, Darmstadt, Germany), trifluoroacetic acid (Thermo Fisher), GalT enzyme (prepared in-house), GalT enzyme monoclonal antibody and HRP-labeled secondary antibody (Sino Biological, Beijing, China, customized), Her2m1 antigen (HER2 domain IV) and Her2m2 antigen (HER2 domain II, murine-derived Fc domain) (prepared in-house), Goat anti-Mouse IgG1 Secondary Antibody, HRP conjugate (Thermo), and HCC1569 (human breast cancer cells) were purchased from the European Collection of Authenticated Cell Cultures (ECACC), and NCI-N87 (human gastric cancer cells) were obtained from the American Type Culture Collection (ATCC). Both cell lines have undergone rigorous testing and have been verified to be free of exogenous contaminants, including mycoplasma. RPMI1640 (Thermo Fisher), fetal bovine serum (FBS) (Thermo Fisher), and an 8CCK8 cell counting kit (DOJINDO Laboratories, Mashiki, Kumamoto, Japan) were used.

### 4.3. Methods

#### 4.3.1. Mispairing Study

The unconjugated antibody was digested using the IdeS enzyme (50:1) at 37 °C for 4–16 h. Trifluoroacetic acid (TFA) was then added to a final concentration of 0.1% to terminate the digestion. This was mixed and prepared for injection. The chromatographic column was a MabPac RP (4 μm, 2.1 × 100 mm). Mobile phase A was 0.1% TFA/99.9% water (*v*/*v*), and mobile phase B was 0.1% TFA/90% acetonitrile/9.9% water (*v*/*v*). The detection wavelength was 214 nm. The column temperature was 70 °C, the injection volume was 5 μL, and the gradient elution was 0.2 mL/min for 17 min. The mispaired percentage content in the unconjugated antibody sample was calculated using the area normalization method.

#### 4.3.2. Average DAR, Unconjugated Antibody, and Drug Payload Distribution

The ADC sample was diluted to 5 mg/mL with ultrapure water. It was then mixed and prepared for injection. The HPLC method: chromatographic column PLRP (1000A, 5 μm, 2.1 × 100 mm), mobile phase A: 0.1% TFA/99.9% water (*v*/*v*), mobile phase B: 0.1% TFA/90% acetonitrile/9.9% water (*v*/*v*). The detection wavelength was 280 nm, the column temperature was 55 °C, the injection volume 5 μL, and the gradient elution was set at 0.4 mL/min for 16 min.

Peak identification was performed using the liquid chromatography method described above, with the column connected to a Waters Xevo G2-XS QTof high-resolution mass spectrometer. The mass spectrometry parameters were the following: positive ion scanning mode, capillary voltage of 3.0 KV, cone voltage of 150 V, ion source temperature of 120 °C, desolvation gas temperature of 500 °C, desolvation gas flow rate of 800 L/h, and mass range of 400–5000 *m*/*z*.

#### 4.3.3. Charge Variants

The ADC sample was diluted to 2.0 mg/mL using a blank buffer. It was then mixed and prepared for injection. Chromatographic column ProPac^TM^ Elite WCX; mobile phase A: 20 mM MES, pH6.8 ± 0.05; mobile phase B: 20 mM MES + 300 mM NaCl, pH6.8 ± 0.05. The flow velocity was 0.5 mL/min, the detection wavelength was 280 nm, the column temperature was 30 ± 5 °C, the injection volume was 10 μL, and the gradient elution was 0.5 mL/min for 15 min.

#### 4.3.4. Conjugation Site

The ADC sample was denatured with a final concentration of 6 mol/L guanidine hydrochloride, reduced with 20 mmol/L dithiothreitol (DTT), and alkylated with 50 mmol/L iodoacetamide (IAM). The sample was then digested with Trypsin (protein: enzyme w:w = 20:1) and incubated at 37 °C for 4 h. After digestion, N-glycosidase (PNGase F) was added to remove N-glycans, and TFA was added to a final concentration of 1% to terminate the digestion. The digested samples before and after deglycosylation were injected separately, the peptide fragments were separated by a reversed-phase chromatographic column (ACQUITY PREMIER Peptide CSH C18), then analyzed by a Xevo G2-XS QTof high-resolution mass spectrometer. The following parameters were used: positive ion scanning mode, capillary voltage of 3.0 KV, cone voltage of 40 V, ion source temperature of 120 °C, desolvation gas temperature of 450 °C, desolvation gas flow rate of 600 L/h, and a mass analysis range of 50–2000 *m*/*z*. The glycopeptides were analyzed according to the detection results of the Waters Xevo G2-XS QTof mass spectrometer and the UNIFI data analysis software (version 1.9.4, Waters), combined with manual confirmation.

#### 4.3.5. Free Drugs

A total of 50 μL of the ADC sample was collected, and 200 μL of dimethyl sulfoxide (DMSO) was added. This was mixed and prepared for injection. The chromatographic column was a reversed-phase PLRP-S chromatographic column, with mobile phase A comprising 0.1% TFA/99.9% water (*v*/*v*); mobile phase B comprising 0.1% TFA/99.9% acetonitrile (*v*/*v*); a detection wavelength of 362 nm; a column temperature of 40 °C; an injection volume of 20 μL; and a gradient elution of 0.4 mL/min for 29 min using three elution gradients to separate the payload, the linker–payload, and the ADC peaks sequentially. The payload and linker–payload were mixed to prepare a standard curve at 80% DMSO/20% water (*v*/*v*) at concentrations of 0.5–25 μg/mL, and the free payload and linker–payload in the sample were quantified separately using the external standard method with the payload and linker–payload mixed standard curve.

The peak identification was performed using the liquid chromatography method described above, with the column connected to a Waters Xevo G2-XS QTof high-resolution mass spectrometer. The peak identification mass spectrometry parameters were the following: a positive ion scanning mode, capillary voltage of 3.0 KV, cone voltage of 40V, ion source temperature of 120 °C, a desolvation gas temperature of 500 °C, a desolvation gas flow rate of 800 L/h, and a mass range of 50–2000 *m*/*z*.

#### 4.3.6. The Residual GalT Enzyme

A double-antibody sandwich ELISA was established using two monoclonal detection antibodies, KNR7A-B0019 (GalT enzyme antibody, rabbit mAb) and KNR7A-S0019-H (GalT enzyme antibody, mouse mAb, HRP), which were generated by immunizing rabbits and mice, respectively, with the GalT enzyme. During the development of these monoclonal antibodies, multiple rounds of screening were performed using GalT as the antigen to ensure high specificity and sensitivity toward the target enzyme. The KNR7A-B0019 was added to the 96-well microplate for coating overnight. After blocking, the sample was incubated. This was followed by the addition of KNR7A-S0019-H. The GalT enzyme in the sample could bind to both KNR7A-B0019 and KNR7A-S0019-H, forming a sandwich complex. 3,3′,5,5′-tetramethylbenzidine (TMB) was added for color development, and the reaction was terminated with a stop solution. The optical density (OD) value was measured at the 450 nm and 650 nm wavelengths using a microplate reader. The OD value was positively correlated with the residual amount of the GalT enzyme in the sample. The residual amount of the GalT enzyme in the sample was calculated by fitting the standard curve with four parameters.

#### 4.3.7. The Relative Binding Activity

The same bridging ELISA was employed for both the unconjugated antibody and its ADC. Microplates were coated with HER2m1 antigen (HER2 domain IV) at 1.5 μg/mL overnight at 4 °C, followed by blocking for 2 h. Serially diluted samples (0.339 ng/mL to 20 μg/mL, 3-fold) were added and incubated for 2 h at room temperature. After washing, 0.3 μg/mL HER2m2 antigen (HER2 domain II) was added, followed by incubation with HRP-conjugated secondary antibody (1:3000). Colorimetric detection was performed using TMB substrate, and the reaction was stopped prior to measuring absorbance at 450 nm with a 650 nm reference.

#### 4.3.8. Biological Activity

HCC1569 (human breast cancer cells, highly expressing HER2 on the cell surface) was used as the target cell. HCC1569 cells were seeded in a 96-well plate at a cell density of 4000 cells/well. This was incubated overnight in a 37 °C and 5% CO_2_ incubator. The supernatant was discarded, and unconjugated antibody or ADC samples with concentration ranges of 0.128 ng/mL–50 μg/mL were added. The cells were incubated for 96 h in a 37 °C and 5% CO_2_ incubator. After incubation, the CCK8 was added, and it was further incubated for 3 h. The OD value was measured at 450 nm and 650 nm wavelengths using a microplate reader.

NCI-N87 (human gastric cancer cells, highly expressing HER2 on the cell surface) was used as the target cell. NCI-N87 cells were seeded in a 96-well plate at cell density of 10,000 cells/well. This was incubated overnight in a 37 °C and 5% CO_2_ incubator. The supernatant was discarded, and unconjugated antibody or ADC samples with a concentration range of 4.572 ng/mL–10 μg/mL were added. The cells were incubated for 72 h in a 37 °C and 5% CO_2_ incubator. After incubation, CCK8 was added and further incubated for 3 h. The OD value was measured at 450 nm and 650 nm wavelengths using a microplate reader.

## Figures and Tables

**Table 1:** Summary of the results of CEX-HPLC collected fraction identification.

Sample Name		Original Sample	AP1	AP2	AP3	MP	BP1	BP2
DAR	Unconjugated antibody%	0.88	0.31	0.29	0.32	0.16	***1.77***	***2.14***
	DAR	3.7	3.7	3.7	3.7	3.8	***3.5***	***3.5***
Intact mass (%)	-K (2), Man5 (2)	1.3	<0.1	0.1	0.1	N.D.	**0.9**	**1.1**
	-K (1), Man5 (2)	0.8	<0.1	<0.1	0.1	0.10%	**3.4**	0.8
	Man5 (2)	N.D.	N.D.	N.D.	N.D.	N.D.	0.2	**3.9**
	Drug (4), -K (2), G0F (2)	63.3	73.6	71.0	79.3	82.6	14.1	25.9
	Drug (4), -K (1), G0F (2)	6.8	N.D.	6.2	N.D.	N.D.	**31.8**	N.D.
	Drug (4), G0F (2)	0.7	N.D.	N.D.	N.D.	N.D.	N.D.	**18.7**
FcγRIIIa affinity (V176, M)		2.07 × 10^−6^	0.95 × 10^−6^	1.91 × 10^−6^	2.40 × 10^−6^	2.20 × 10^−6^	1.36 × 10^−6^	0.71 × 10^−6^
FcγRIIIa affinity (F176, M)		2.55 × 10^−6^	1.19 × 10^−6^	3.53 × 10^−6^	6.03 × 10^−6^	3.21 × 10^−6^	3.46 × 10^−6^	1.61 × 10^−6^
C1q affinity (KD, M)		1.48 × 10^−7^	1.90 × 10^−7^	1.92 × 10^−7^	1.75 × 10^−7^	1.87 × 10^−7^	1.59 × 10^−7^	1.96 × 10^−7^
FcRn affinity (KD, M)		4.29 × 10^−8^	5.67 × 10^−8^	5.85 × 10^−8^	5.36 × 10^−8^	5.65 × 10^−8^	4.03 × 10^−8^	3.38 × 10^−8^
Relative binding activity (%)		107	86	92	93	87	96	87
Cell proliferation inhibition activity (%)		98	85	107	98	110	94	91
DSF	Tm1 (°C)	61.62	63.34	63.46	63.65	63.96	62.89	62.94
	Tm2 (°C)	85.87	85.65	85.75	85.8	85.95	85.77	85.78
N-deamidation	LC:N30	3.5	***25.0***	***16.8***	***8.4***	1.7	2.4	2.2
	HC:N55	0.6	***3.0***	0.1	0.1	0.5	0.6	0.6
C-terminal P amidation		7.7	4.1	4.3	3.5	1.1	**21.7**	**29.6**
C-terminal K deletion		87.5	94.9	94.5	95.2	98.3	**57.6**	**46.7**
D-cyclization	HC:D102	2.0	1.5	1.4	1.3	1.5	***2.9***	***9.4***
D-isomer	HC:D102	4.2	3.2	2.1	2.1	1.2	***7.9***	***13.4***

Note: Bold and italicized results indicate the components detected in the acidic and basic peak fractions that differ from those in the main peak.

**Table 2:** CQA risk assessment and scoring employed in this study.

Quality Attribute	Test Item	Severity		Possibility		Risk Score	Risk Level	Attribute Type
		Score	Basis (Literature or Animal/Human Trial Evidence)	Score	Basis (Data Analysis Results)			
**Mispairing**	RP-HPLC	5	Heavy chain/heavy chain homologous mispairing, and the activity and stability of the bispecific antibody may be affected [16].	5	Consistent between batches. No increasing trend in the long-term stability and an unconjugated antibody.	25	Medium risk	Non-CQA
**Conjugation site**	LC-MS/MS	5	Site-specific conjugation technology is used. The conjugation site of the linker–payload is the N-glycosylation site in the Fc region of this product.	1	Non-site-specific conjugation of the linker–payload has not been found at other sites.	5	Low risk	Non-CQA
**DAR**	RP-HPLC	7	The efficacy and half-life of molecules with different loading capacities may differ, and there may be safety issues [17].—**Safety, efficacy, PK**	5	Consistent between batches. No increasing trend in the long-term stability. The conjugation distribution is affected by the N-glycan profile, and the uniformity of the N-glycan profile is influenced by the cell culture process, with a moderate possibility of affecting the DAR.	35	High risk	***CQA***
**Unconjugated antibody**	RP-HPLC	7	The unconjugated antibody occupies the target site, thereby hindering the effective binding of ADC and affecting the therapeutic efficacy; —**Efficacy** The unconjugated antibody has a half-life that is different from that of ADC [18];—**PK** The unconjugated antibody has higher safety than ADC and is without cytotoxic effects.—**Safety**	3		21	Medium risk	Non-CQA
**Drug loading distribution**	RP-HPLC	7	Different drug loading distributions can affect the average DAR, thereby influencing the pharmacological efficacy and half-life, and these may lead to safety issues [17,18]—**Safety, Efficacy, PK**	5		35	High risk	***CQA***
**Residual** **free drugs**	RP-HPLC	7	The toxin in this project can bind to the topoisomerase I-DNA cleavable complex, thereby inducing double-strand DNA breaks, ultimately leading to cancer cell apoptosis [19].—**Efficacy** The payload has a very small molecular weight, reducing the risk of immunogenicity.—**Immunogenicity and Safety** Free toxins do not have targeted cytotoxic effects;—**Efficacy** The payload is lipophilic and has a short half-life in the blood. Animal experiments show that it has a very short half-life in the circulatory system; [20]—**PK** Additionally, the payload also has a bystander cytotoxic effect on adjacent cells [21].—**Efficacy**	5	The batches are basically consistent. No increasing trend in long-term stability. The possibility of free drugs that affects safety is medium.	35	High risk	***CQA***
**Residual** **GalT enzyme**	ELISA	7	The enzyme needed during the conjugation process is β-1,4-galactosyltransferase (GalT), with a Y289L mutation in the sequence to enhance the transferase activity toward GalNAz. The safety and immunogenicity of the mutated enzyme after intravenous administration into humans are unknown.	3	The results between batches are all below 5 ppm, and this residual level has not shown obvious safety, immunogenicity, or toxicity-related phenomena in clinical studies.	21	Medium risk	Non-CQA
**Deamidation**	CEX-HPLC	5	Deamidation is common in human-derived proteins and recombinant proteins. N-deamidation-sensitive sites N30 (light chain LC) and N55 (heavy chain II, i.e., HC-Tmab) are both located in the CDR region. Literature [22] from Roche on the deamidation characterization strategy of HER2 antibodies mentions that N30 in the light chain LC does not affect the biological activity of HER2, while N55 in the heavy chain HC may naturally undergo deamidation in vivo, indicating a low in vivo safety risk and no impact on biological function.	3	After collection and identification of the CEX fractions, different proportions of N30 and N55 deamidation were found in several acidic peak fractions. The relative binding activity, antigen affinity, and proliferation inhibition activity of several fractions showed no significant differences compared to the main peak.	15	Medium risk	Non-CQA
**Aspartic acid isomerization**	CEX-HPLC	7	D102 isomerization of Trastuzumab may affect its in vitro cell activity and potentially affect drug efficacy [23].	3	After collection and identification of the CEX fractions, different proportions of D102 isomerization were found in several basic peak fractions. The relative binding activity, antigen affinity, and proliferation inhibition activity of several fractions showed no significant differences compared to the main peak.	21	Medium risk	Non-CQA
**Relative binding activity**	Bridging ELISA	***Obligatory CQA***						
**Biological activity**	Cell proliferation inhibition activity	***Obligatory CQA***						

## Data Availability

The data presented in this study are available upon request from the corresponding author. The data are not publicly available due to restrictions related to product registration and ongoing patent applications.

## Supplementary Materials

The following supporting information can be downloaded at: https://www.mdpi.com/article/10.3390/ph19091495/s1, Figure S1. Reinjection WCX-HPLC chromatograms of the collected fractions. Figure S2. Mass spectra of the linker-payload conjugation site-containing peptide (EEQYNSTYR) before and after deglycosylation. (a) MS spectrum of the peptide before deglycosylation. (b) MS spectrum of the peptide after deglycosylation. (c) MS/MS spectrum of the peptide before deglycosylation. (d) MS/MS spectrum of the peptide after deglycosylation. Table S1. Summary of mispairing method validation results. Table S2. Summary of DAR method validation results. Table S3. Summary of charge variants method validation results. Table S4. Purity of the collected fractions. Table S5. Mass errors of the conjugation site-containing peptide (EEQYNSTYR) before and after deglycosylation. Table S6. Quantitative analysis of conjugation occupancy based on MS spectral data. Table S7. Summary of free drugs method validation results. Table S8. Summary of residual GalT enzyme method validation results. Table S9. Summary of relative binding activity method validation results. Table S10. Summary of biological activity method validation results.

## Abbreviations

The following abbreviations are used in this manuscript:

ADC	Antibody–Drug Conjugate
BsADC	Bispecific Antibody–Drug Conjugate
CQA	Critical Quality Attribute
HER2	Human Epidermal Growth Factor Receptor 2
DAR	Drug-to-Antibody Ratio
LC-MS	Liquid Chromatography–Mass Spectrometry
RP-HPLC	Reversed-Phase High-Performance Liquid Chromatography
CEX-	Cation-Exchange Chromatography
*m*/*z*	Mass-to-Charge Ratio
LOQ	Limit of Quantitation
L-P	Linker–Payload
r^2^	Coefficient of Determination
ELISA	Enzyme-Linked Immunosorbent Assay
F(ab′)2	Fragment antigen-binding (divalent)
Fc/2	Half-Fc fragment
RSD	Relative Standard Deviation
DSF	Differential Scanning Fluorimetry
FcRn	Neonatal Fc Receptor
FcγRIII	Fc Gamma Receptor III
C1q	Complement Component 1q
IC_50_	Half-Maximal Inhibitory Concentration
MoA	Mechanism of Action
ADCC	Antibody-Dependent Cell-Mediated Cytotoxicity
CDC	Complement-Dependent Cytotoxicity
