# Supplementary material for: Critical Quality Attribute Investigation and Analytical Method Establishment for Glyco-Site-Specific Anti-HER2 Bispecific ADC
Source: Pharmaceuticals (Basel). 2026 Sep 20;19(9):1495. doi: 10.3390/ph19091495 (PMC13610955; doi:10.3390/ph19091495)
Supplement: Supplementary file 1 [file pharmaceuticals-19-01495-s001.zip › pharmaceuticals-4528843-supplementary.pdf]

SUPPLEMENTARY MATERIALS

Table S1. Summary of mispairing method validation results.

Validation Parameter	Mispairing			
**Speicificity**	The formulation buffer shows no interfering peaks in the integration region.			
**Precision**	Heavy Chain I Impurity		Heavy Chain II Impurity	
	**Repeatability %CV (3 injections)**	**Intermediate precision %CV (6 injections, different analysts,days, instruments, and columns)**	**Repeatability %CV (3 injections)**	**Intermediate precision %CV (6 injections, different analysts,days, instruments, and columns)**
Low（HC II: 0.5%, HC I: 8.0%）	0.8	1.1	4.1	4.1
Middle（HC II: 1.3%, HC I: 16.0%）	1.4	1.2	3.3	3.0
High（HC II: 2.0%, HC I: 24.0%）	0.2	1.5	0.7	1.5
**Linearity and range (5 levels)**				
**r^2**	0.9998		0.9969	
**Range**	0.5%-24.0%		0.5%-2.1%	
**Accuracy (3 levels, 3 replicates each)**	**% Recovery**	**% CV**	**% Recovery**	**% CV**
Low（HC II: 0.5%, HC I: 8.0%）	100.2~101.6	0.8	89.3~96.9	4.1
Middle（HC II: 1.3%, HC I: 16.0%）	99.5~102.0	1.4	99.6~106.1	3.3
High（HC II: 2.0%, HC I: 24.0%）	99.6~100.0	0.2	96.9~98.6	0.7
**LOQ**	0.5%		0.5%	
**Robustness**	Test solution stable at 8°C for 48 h in autosampler.			

Table S2. Summary of DAR method validation results.

**Validation Parameter**	**Average DAR, unconjugated antibody, and drug conjugation distribution**					
**Speicificity**	The formulation buffer shows no interfering peaks in the integration region,<br>Unconjugated antibody elutes at D0,<br>Free drug shows no interference in the D0–D5 elution region.					
**Precision（3 levels）**	**Average DAR**		**D0**		**D4**	
			**% CV**			
Repeatability（3 injections/ level）	0.2		1.9		2.1	
Intermediate precision (different analysts, days, instruments, and columns，6 injections/level）	0.3		2.3		2.2	
**Linearity and range (5 levels)**						
**r²**	N/A		0.9996		0.9998	
**Range**	1.5~4.0		0.3%-10.9%		≥29.1%	
**Accuracy (3 levels, 3 replicates each)**	**% Recovery**	**% CV**	**% Recovery**	**% CV**	**% Recovery**	**% CV**
Low（2 mg/ml）	99.8~99.9	0.1	98.0~104.6	3.4	102.5~104.0	0.80%
Middle（5.0 mg/ml）	100.0	0.0	99.3~101.1	1.0	98.8~102.2	1.9
High（12.5 mg/ml）	100.3	0.0	100.7~101.9	0.6	98.7~102.0	1.8
**LOQ**	N/A		0.30%		N/A	
**Robustness**	Test solution stable at 8°C for 48 h in autosampler.					
Notes：N/A=Not Appliable						

Table S3. Summary of charge variants method validation results.

Validation Parameter	Charge variants					
**Speicificity**	The formulation buffer shows no interfering peaks in the integration region,<br>Thermal stress sample shows a trend of change with increasing degradation.					
**Precision (3 levels)**	**Acid species**		**Main peak**		**Basic species**	
	**% CV**					
Repeatability (3 replicates each)	2.9		1.1		4.1	
Intermediate precision （different analysts, days, instruments, and columns，6 injections/level）	2.6		1		3	
**Linearity and range (5 levels)**						
**r²**	1.0000		1.0000		1.0000	
**Range**	0.4%-38.2%		≥1.5%		0.6%-37.5%	
**Accuracy (3 levels, 3 replicates each)**	**% Recovery**	**% CV**	**% Recovery**	**% CV**	**% Recovery**	**% CV**
Low（0.05 mg/ml）	95.9~97.8	1.0	98.5~100.3	1.0	100.7~105.8	2.80%
Middle（2.0 mg/ml）	100.0	0.0	99.9~100.1	0.1	99.7~100.2	0.3
High（4.0 mg/ml）	100.4~100.5	0.1	99.7~99.8	0.1	100.1~100.5	0.2
**LOQ**	0.40%		1.50%		0.60%	
**Robustness**	Test solution stable at 8°C for 48 h in autosampler.					

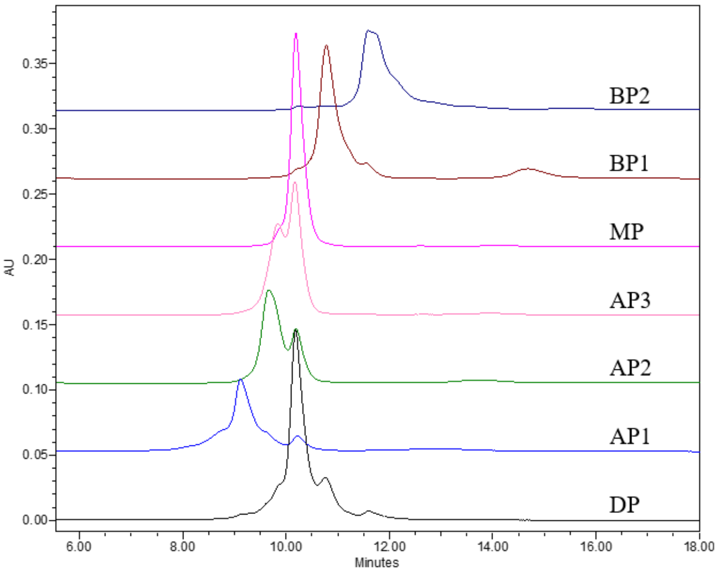

Figure S1 Reinjection WCX-HPLC chromatograms of the collected fractions.

Table S4. Purity of the collected fractions.

Fraction ID	**AP1**	**AP2**	**AP3**	**MP**	**BP1**	**BP2**
Reinjection purity / %	72.3	64	44	89.8	77.8	92.2

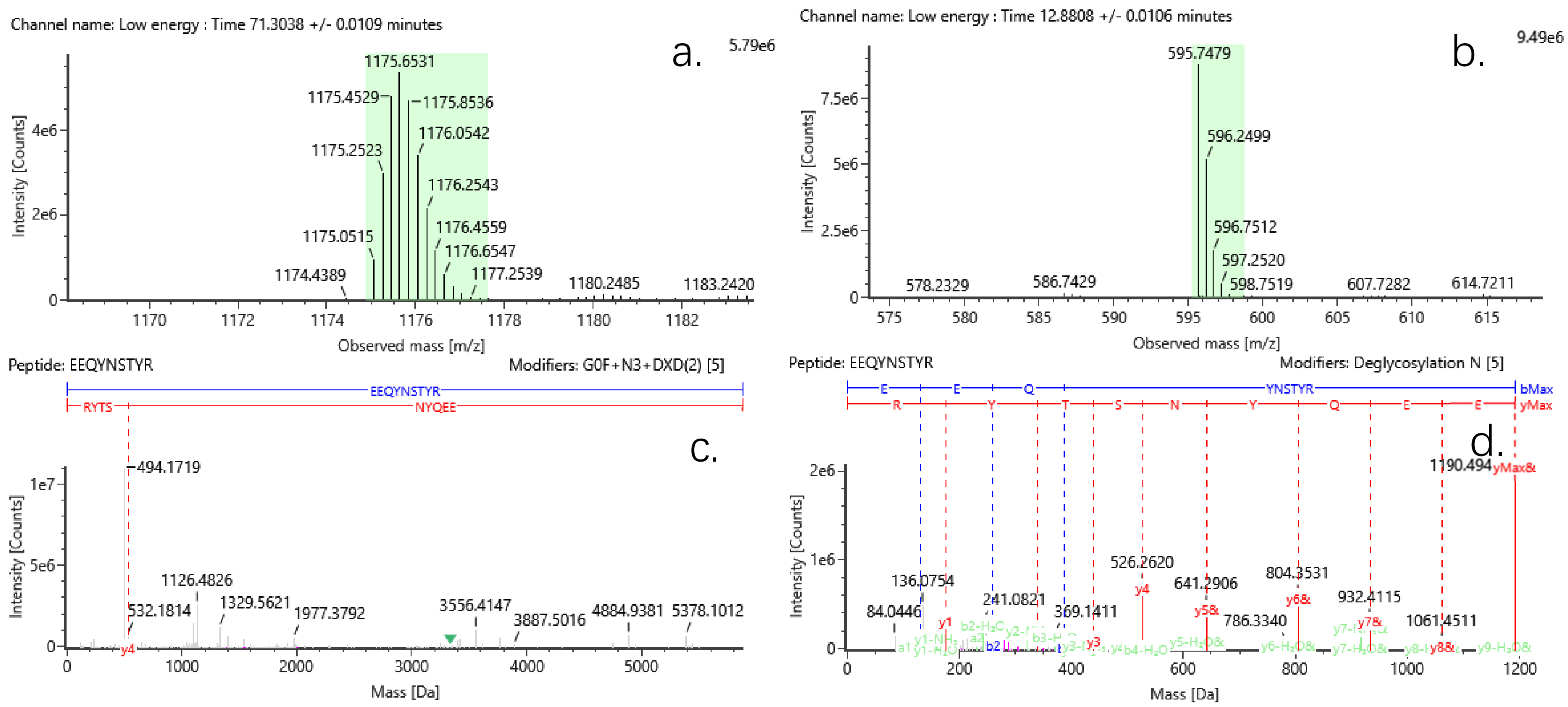

Figure S2. Mass spectra of the linker-payload conjugation site-containing peptide (EEQYNSTYR) before and after deglycosylation. (a) MS spectrum of the peptide before deglycosylation. (b) MS spectrum of the peptide after deglycosylation. (c) MS/MS spectrum of the peptide before deglycosylation. (d) MS/MS spectrum of the peptide after deglycosylation.

Table S5. Mass errors of the conjugation site-containing peptide (EEQYNSTYR) before and after deglycosylation.

Peptide	Modifiers	Expected m/z	Observed m/z	Charge	Expected mass (Da)	Observed mass (Da)	Mass error (ppm)
EEQYNSTYR	Deglycosylation N [5]	595.7517	595.7480	2	1190.4960	1190.4888	-6.1
EEQYNSTYR	G0F+2*LP [5]	1175.0717	1175.0516	5	5871.32916	5871.2290	-17.1

Table S6. Quantitative analysis of conjugation occupancy based on MS spectral data.

Label	Peptide	Modifiers	Observed RT (min)	Response	Percentage
**P:T24**	EEQYNSTYR	/	11.46	135868	0.17%
**P:T24&**	EEQYNSTYR	Glycosylation Man5 N [5]	11.74	558275	0.68%
**P:T24&**	EEQYNSTYR	Glycosylation G2F N [5]	12.58	71807	0.09%
**P:T24&**	EEQYNSTYR	G1F+N3+LP(1) [5]	49.52	774783	**3.08%**
**P:T24&**	EEQYNSTYR	G1F+N3+ LP (1) [5]	49.77	1754306	
**P:T24&**	EEQYNSTYR	G0F+N3+ LP (2) [5]	70.98	18761180	**95.99%**
**P:T24&**	EEQYNSTYR	G0F+N3+ LP (2) [5]	71.30	40639208	
**P:T24&**	EEQYNSTYR	G0F+N3+ LP (2) [5]	71.53	19517716	

SUM (EEQYNSTYR G0F/G1F+N3+LP) =99.1%

Table S7. Summary of free drugs method validation results.

**Validation Parameter**	**Free drug**			
**Speicificity**	Formulation Buffer and 80% DMSO shows no interfering peaks in the integration region, Distinct degradation peaks observed in thermal stress sample vs. control, demonstrating stability-indicating(see Figure 3).			
	Payload		Linker-Payload	
**Intermediate precision**（different analysts, days, instruments, and columns，6 injections/level）	**% CV**			
Low（0.5 ug/ml）	1.1		1.7	
Middle（5.0 mg/ml）	1.3		1.2	
High（25.0 mg/ml）	1.7		1.1	
**Linearity and range (5 levels)**				
**r^2**	1.0000		0.9999	
**Range**	0.5μg/ml~25.0μg/ml		0.5μg/ml~25.0μg/ml	
**Accuracy (3 levels, 3 replicates each)**	**% Recovery**	**% CV**	**% Recovery**	**% CV**
Low（0.5 ug/ml）	98~105	3.6	91~94	1.9
Middle（5.0 mg/ml）	103	0	103~104	0.6
High（25.0 mg/ml）	103	0	102	0
**LOQ**	0.5μg/ml		0.5μg/ml	
**Robustness**	Test solution stable at 8°C for 29 h in autosampler.			

Table S8. Summary of residual GalT enzyme method validation results.

Validation Parameter	Residual GalT enzyme	
**Speicificity**	Formulation Buffer shows no interfering peaks in the integration region	
**Precision**	**Repeatability %CV (3 replicates)**	**Intermediate precision %CV （2 analysts on 2 days，6 replicates）**
Low（0.5 ng/ml）	1	2
Middle（1 ng/ml）	1	2
High（3 ng/ml）	3	4
**Linearity and range (5 levels)**		
**r^2**	0.9998	
**Range**	0.25 ng/ml~5 ng/ml	
**Accuracy (3 levels, 3 replicates each)**	**% Recovery**	**% CV**
Low（0.5 ng/ml）	87	1
Middle（1 ng/ml）	89	1
High（3 ng/ml）	94	3
**LOQ**	0.25 ng/ml	

Table S9. Summary of relative binding activity method validation results.

Validation Parameter	Relative Binding Activity	
**Speicificity**	Formulation buffer shows no typical S-shaped curve, Relative binding activity of thermal stress sample changed with increasing degradation.	
**Precision（3 levels）**	**Repeatability %CV (3 replicates)**	**Intermediate precision %CV（2 analysts on 2 days，6 replicates）**
Potency level（50%）	4	6
Potency level（100%）	8	6
Potency level（201%）	4	5
**Linearity and range (5 levels)**		
**r²**	0.999	
**Range**	50%~201%	
**Accuracy (3 levels, 3 replicates each)**	**RB/%**	**GCV/%**
Potency level（50%）	-4~2	4
Potency level（100%）	-9~4	8
Potency level（201%）	-4~1	4

Table S10. Summary of biological activity method validation results.

Validation Parameter	Biological Activity	
**Speicificity**	Formulation buffer shows no typical S-shaped curve, Biological activity of thermal stress sample changed with increasing degradation.	
**Precision（3 levels）**	**Repeatability %CV (3 replicates)**	**Intermediate precision %CV（2 analysts on 2 days，6 replicates）**
Potency level（50%）	4	6
Potency level（100%）	4	5
Potency level（201%）	4	6
**Linearity and range (5 levels)**		
**r^2**	0.9965	
**Range**	50%~201%	
**Accuracy (3 levels, 3 replicates each)**	**% RB**	**% GCV**
Potency level（50%）	-2~0	4
Potency level（100%）	5~10	4
Potency level（201%）	-2~1	4
